# Synergetic strengthening of layered steel sheet investigated using an *in situ* neutron diffraction tensile test

**DOI:** 10.1038/s41598-019-43369-2

**Published:** 2019-05-02

**Authors:** Jung Gi Kim, Jae Wung Bae, Jeong Min Park, Wanchuck Woo, Stefanus Harjo, Kwang-Geun Chin, Sunghak Lee, Hyoung Seop Kim

**Affiliations:** 1 Department of Materials Science and Engineering, Pohang University of Science and Technology (POSTECH), Pohang, 37673 Korea; 2 Korea Atomic Energy Research Institute (KAERI), Daejeon, 34057 Republic of Korea; 3 J-PARC Center, Japan Atomic Energy Agency, Ibaraki, 319-1195 Japan; 4 Graduate Institute of Ferrous Technology, Pohang University of Science and Technology (POSTECH), Pohang, 37673 Korea; 5 Center for High Entropy Alloys, Pohang University of Science and Technology (POSTECH), Pohang, 37673 Korea

**Keywords:** Metals and alloys, Mechanical properties

## Abstract

Synergetic strengthening induced by plastic strain incompatibility at the interface, and the resulting extra geometrically necessary dislocations (GNDs) generated during plastic deformation, were investigated to understand the origin of extra strength in heterogeneous structured (HS) materials. The mechanism of extra GND generation in twinning-induced plasticity (TWIP)-interstitial free (IF) steel layered sheet was quantitatively analyzed by conducting *in situ* neutron scattering tensile test. Load partitioning due to the different mechanical properties between the TWIP-steel core and IF-steel sheath at the TWIP/IF interface was observed during the *in situ* tensile testing. Because of the plastic strain incompatibility from load partitioning, extra GNDs are generated and saturate during tensile deformation. The extra GNDs can be correlated with the back-stress evolution of the HS materials, which contributes to the strength of layered materials. Because of the back-stress evolution caused by load partitioning, the strength of TWIP-IF layered steel is higher than the strength estimated by the rule-of-mixtures. This finding offers a mechanism by which extra GNDs are generated during load partitioning and shows how they contribute to the mechanical properties of HS materials.

## Introduction

During the last decade, heterogeneous structured (HS) materials (i.e., layered structures^1–3^, bimodal structures^4,5^, and gradient structures^6,7^) have been extensively developed due to their outstanding strength-ductility combination. Such outstanding mechanical properties of HS materials provide applicability to advanced structural materials. Among the HS materials, the steel-based layer materials have been studied for the next-generation automotive steel sheets. In previous research, Bouaziz *et al*. reported on the combination of twinning-induced plasticity (TWIP) and martensitic steel^8^, while Koseki *et al*. developed stainless-martensitic multilayered steel^9^. Both these two-layered steel sheets were manufactured simply using a roll-bonding process and achieved high strength with moderate ductility. Moreover, in the current authors’ previous research, the TWIP-cored three-layer steel sheet roll-bonded with mild steels (i.e., low carbon (LC) and interstitial free (IF) steels) not only represents high strength which is larger than the estimated strength by the simple rule-of-mixtures^10^ but also suppresses deformation instability^11^. Previous reports successfully show that the outstanding properties of layered materials occur because certain mechanisms that operate at the interfaces between the layered materials add extra strength.

Synergetic strengthening at the interfaces of layered sheets originates from the accumulation of dislocations and biaxial stress state due to plastic strain incompatibility between hard and soft phases during plastic deformation^12^. To sustain geometric compatibility and to relieve this plastic strain incompatibility, back-stress evolves and generates extra geometrically necessary dislocations (GNDs). These additional GNDs result in the strength enhancement of HS materials so that they are stronger than the sum of the strength of separate components by the simple rule-of-mixtures^13^. Although this synergetic strengthening provides additional strength and positive effects to the HS materials, the evolution of the extra dislocations during plastic deformation, has not yet been explained quantitatively.

To investigate the extra generation of dislocations, some researchers measured the GND density by conducting electron backscattering diffraction (EBSD) analysis, or calculated the dislocation density using X-ray diffraction peak profile analysis. Ma *et al*. represented the GND density distribution of copper/brass laminated materials by EBSD mapping and found that the GND density increases with an increase in the number of copper/brass interfaces^1^. Although the accumulated GNDs are observed near the interface region, the increment of GNDs was within the standard deviation due to the locally generated GNDs from the gradient of non-uniform strain, and from the crystallographic orientation. Moreover, in the authors’ previous work, the dislocation density increment of the TWIP-steel core in TWIP-cored three-layer steel sheets was quantified using X-ray peak profile analysis^11^. However, this exhibited large deviation due to the limited measurement volume during X-ray diffraction analysis. Therefore, other experimental techniques should be considered to quantify the extra-dislocation density for the synergetic strengthening of materials.

The *in situ* neutron diffraction tensile test is an efficient method for investigating the deformation mechanisms of multi-layered materials due to the large penetration depth of neutrons^14^. This allows sufficient measurement volume during testing, and provides representability and reliability of the results. From the neutron diffraction analysis, load partitioning and deformation mechanism of the multi-layered materials can be revealed by measuring the elastic lattice strain (*ε*_hkl_) and peak broadening changes during tensile deformation. The *ε*_hkl_ of materials provides the residual stress and microscopic deformation state during tensile deformation^15^. Here, peak broadening is related to the internal defects (i.e., grain boundaries, dislocations, and deformation twins) of the materials^16^. Because the back-stress starts to evolve from an early stage of deformation^17^, investigation of the load partitioning in layered materials from the initial state to fracture becomes an important issue.

In this study, the role of synergetic strengthening on the generation of extra dislocations and the mechanical properties of TWIP-IF layered steel sheet was investigated using an *in situ* neutron diffraction tensile test. To track the changes in *ε*_hkl_ as the elongation increases, diffraction peaks were measured every 50 MPa spacing in the elastic deformation stage, and 5% elongation spacing in the plastic deformation stage. To quantify the dislocation density of the tensile deformed layered steel sheet, peak broadening was calculated using the modified Williamson-Hall plot. To compare with the evolution of load partitioning and extra-dislocation density from the neutron diffraction analysis, conventional tensile tests were conducted.

**Figure 1:**
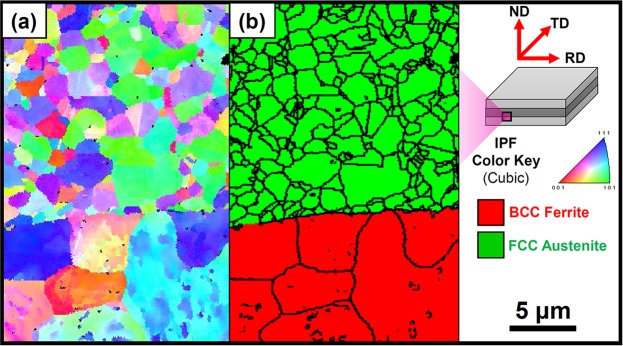
EBSD analysis results of the interfacial region of TWIP-IF layered steel sheet. (**a**) Inverse pole figure and (**b**) phase distribution map.

**Figure 2:**
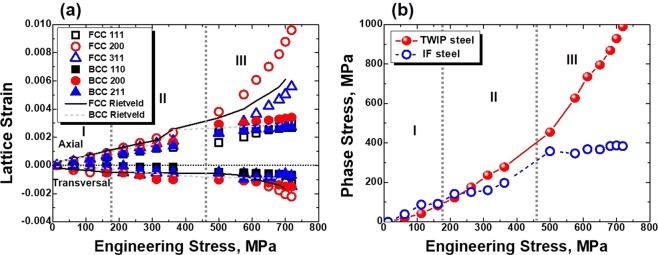
(**a**) Axial and transversal *ε*_hkl_ of TWIP-IF layered steel with the applied stress and (**b**) phase stress evolution during the tensile deformation.

Figure 1 represents the initial microstructure in the interfacial region of the layered steel. Because the TWIP-IF steel layer has a clear austenite-ferrite interface, the other factors (i.e., interface delamination, diffusion, phase transformation, etc.) are excluded from this study. Figure 2(a) is a plot of the *ε*_hkl_ of TWIP-IF layered steel as the applied load increases. The *ε*_hkl_ of materials can be calculated by the diffraction peak shift. Because the peak position of materials shifts as the amount of stress increases, *ε*_hkl_ can be calculated from the deviation of lattice spacing of the specific hkl-crystal plane as follows^18^:

1 $${\varepsilon }_{hkl}=\frac{{d}_{hkl}-{d}_{hkl}^{0}}{{d}_{hkl}^{0}},$$

where *d*_hkl_ is the lattice spacing of the tensile deformed specimen and *d*_hkl_^0^ is the lattice spacing of the initial specimen. Because of the different properties between TWIP steel and IF steel, three-step partitioning is observed as follows. (i) In STAGE 1, both *ε*_hkl_^FCC^ and *ε*_hkl_^BCC^ increase simultaneously due to the elastic deformation of the TWIP-steel core and IF-steel sheath. (ii) In STAGE 2, the IF-steel sheath starts to exhibit plastic deformation and the slope of *ε*_hkl_^BCC^ slightly decreases as the applied load increases. (iii) In STAGE 3, both TWIP-steel core and IF-steel sheath plastically deform and the slopes of *ε*_hkl_ changes with increase of the applied force. Both TWIP-steel core and IF-steel sheath are constrained by each other. In addition, the *ε*_hkl_ slope change at STAGE 3 is dependent on the slip systems and elastic anisotropy of materials^19^. In the FCC materials, the (111)_FCC_ lattice plane acts as (111) <11-2> slip systems and the applied load can be relieved due to the dislocation slip. This means that *ε*_111_^FCC^ is relieved by a dislocation slip after yielding and has lower *ε*_hkl_ than in the other planes. Meanwhile, the (200)_FCC_ and (311)_FCC_ planes show tendency to be stiffer than the (111)_FCC_ plane due to their limited slip systems^20^. In BCC materials, however, all the lattice planes (except the (200)_BCC_ plane) are able to achieve dislocation slip due to the pencil glide system^21^. Because the dislocation slip relieves *ε*_hkl_ during plastic deformation, *ε*_hkl_^BCC^ increases slowly compared with the case in STAGE 1. Similar to the IF-steel sheath in STAGE 2, the interfacial region of the TWIP-steel core is also constrained by the IF-steel sheath.

From the axial and transversal *ε*_hkl_, stress evolution in each phase can be calculated by using Hooke’s Law^22,23^. For a phase stress calculation, *E* and *ν* for TWIP-IF layered steel are provided in Table 1 ^24,25^. The average axial and transversal *ε* are obtained from the Rietveld refinement, see Fig. 2(a). Figure 2(b) represents the phase stress evolution of TWIP-IF layered steel during the tensile deformation, showing clear load partitioning with the applied stress. Because the IF-steel sheath represents lower strength than the TWIP-steel core, the BCC phase stress starts to deviate from linearity at STAGE 2 while the FCC phase stress keeps the linearity until the yield strength of layered steel and increases after the yield strength. Similar lattice strain or stress deviations can be observed in the other dual phase and transformation induced plasticity steels containing hard austenite and soft ferrite phases^26,27^. In the multi-phase steels, such partitioning can induce lattice strain deviation from the rule-of-mixtures due to the stress transfer interruption by the microstructural complexity. However, in the layered steel, the simple structure allows easier stress transfer between soft and hard phases than the multi-phase steels that prevent severe stress concentration in the soft phase^14^. Therefore, the layer structured steel can easily maintain strength-ductility balance in comparison with multi-phase steels. Meanwhile, plastic strain incompatibility remains some at the interfacial region though the severe stress concentration has been relieved^1^. To cover this strain incompatibility, GNDs are accumulated in the interface and these GNDs can be treated as an internal defect which induces the diffraction peak broadening.

**Table 1:** The values of *E* and *ν* used for stress calculations^24,25^.

	*E*, MPa	*ν*
TWIP steel	156,000	0.3
IF steel	136,500	0.3

The dislocation density of materials can be obtained from the broadening of peak profiles using peak-profile-analysis methodology. The modified Williamson-Hall plot is a typical peak-profile analysis method for evaluating crystallite size and dislocation density based on the full width at half-maximum (FWHM) of the diffraction peaks^28^:

2 $${\rm{\Delta }}K=\frac{0.9}{D}+{(\frac{\pi {A}^{2}{b}^{2}}{2})}^{\frac{1}{2}}{\rho }^{\frac{1}{2}}(K{C}^{\frac{1}{2}})+O({K}^{2}C),$$

where Δ*K* is 2 cos *θ*(Δ*θ*)/*λ* (FWHM), *K* is 2 sin *θ*/*λ* (peak position), *θ* is the diffraction angle, *λ* is the wavelength, *A* is the constant determined by the effective outer cut-off radius of dislocations and *b* is the Burgers vector. In this research, the b of TWIP steel is 0.2553 nm and b of IF steel is 0.248 nm. *C* is the contrast factor of dislocations which can be calculated as follows^29^:

3 $$C={C}_{h00}(1-q(\frac{{h}^{2}{k}^{2}+{k}^{2}{l}^{2}+{l}^{2}{h}^{2}}{{({h}^{2}+{k}^{2}+{l}^{2})}^{2}})),$$

where *C*_h00_ values were calculated by the elastic constant of *C*_11_, *C*_22_, and *C*_44_ of TWIP and IF steels, *q* is related to the nature of the screw or edge dislocations. The Eq. (2) represents that *KC*^1/2^ is the appropriate scaling factor of the FWHM of line profiles if the dislocation is the main factor of strain in crystal. From the modified Williamsom-Hall plot, we can obtain the Δ*K*-*KC*^1/2^ slope (*m*), and this can be correlated with the dislocation density (*ρ*) of materials as follows:

4 $$\rho =\frac{2}{\pi {A}^{2}{b}^{2}}{m}^{2}.$$

**Figure 3:**
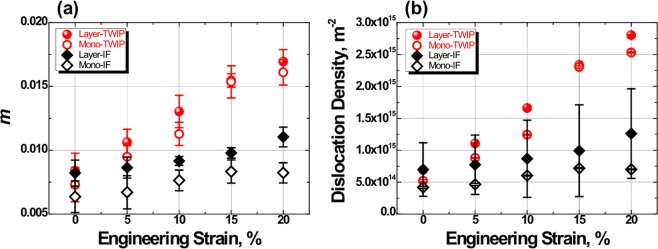
(**a**) *m* and (**b**) dislocation density changes of the tensile deformed TWIP, IF, and TWIP-IF layered steels. *m* of materials was fitted from the modified Williamson-Hall plots (see Fig. S3).

Figure S1 represents the modified Williamson-Hall plots of the TWIP, IF, and TWIP-IF layered steels (See Fig. S1), and the *m* can be obtained by linear fitting procedure with these points. Figure 3 shows the *m* and dislocation density changes of the TWIP, IF, and TWIP-IF layered steels with increase of the tensile strain. The *m* of TWIP-IF layered steel is larger than in monolithic materials, and the increased *m* results in an increase of the dislocation density, as shown in Fig. 3(b). In TWIP steel, however, twin boundaries also contribute to the peak broadening and this additional peak broadening overestimates the calculated dislocation density of the TWIP steel^30^. However, it is clear that large *m* values of the TWIP-steel core and IF-steel sheath mean the total internal defects (i.e. dislocations and twins) of the layered TWIP-IF layered steel is larger than those in monolithic materials^16^. The evolution of the extra-dislocation density in the TWIP-IF layered steel sheet can be explained in two ways. (i) Dislocations accumulate in the TWIP/IF interface during load partitioning. This dislocation accumulation stops after sufficient generation of dislocations in the interface. Therefore, the extra-dislocation density evolves in the early plastic deformation and saturates in the later deformation stage. (ii) In the TWIP-steel core, additional dislocation-twin interactions occur due to the extra-twin boundaries at the interface region^31^. The extra-twin boundaries in the TWIP steel-core is originated from the additional lateral compressive stress due to the IF steel-sheath shrinkage^6,12^.

**Figure 4:**
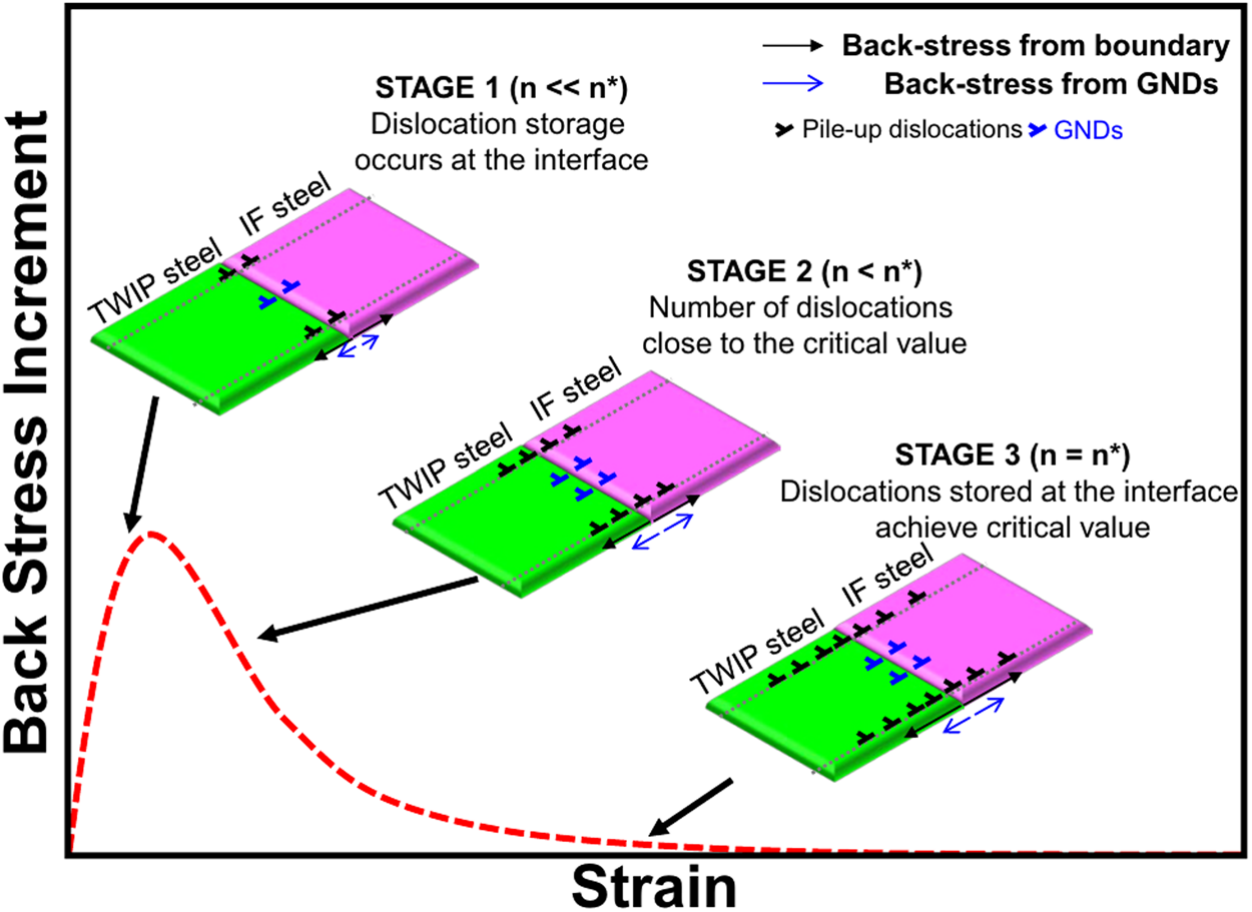
Relationship between dislocation evolution and the back-stress evolution at the TWIP-IF interface during tensile deformation.

**Figure 5:**
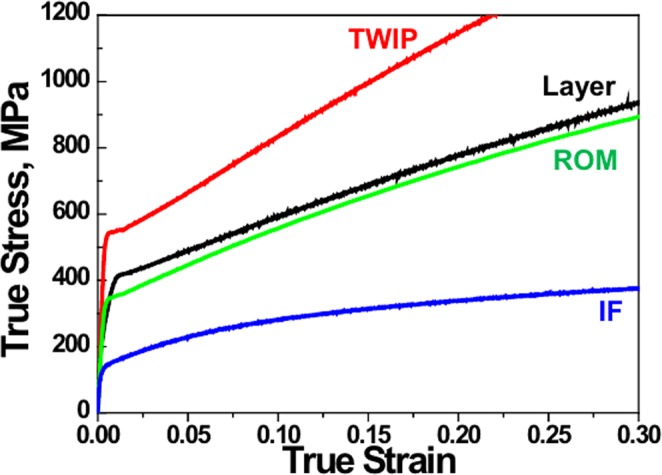
True stress-strain curves of the TWIP, IF, and TWIP-IF layered steels. The stress-strain curves were obtained from conventional tensile tests.

The evolution of the extra-dislocation density of the TWIP-IF layered steel can be correlated with the back-stress evolution, in that the result shows that back-stress evolves in the early stage of plastic deformation and does not affect the larger strain^17^. Figure 4 represents the relationship between dislocations and back-stress evolution at the TWIP-IF interface during plastic deformation. In STAGE 1, dislocation storage occurs at the interface and back-stress evolves due to long-range interaction through mobile dislocations^32^. Because dislocation generation originates from dislocation slip and GND accumulation, both interface boundary and GNDs contribute to back-stress^33,34^. In STAGE 2, the number of dislocations (n) is close to the critical value (n*) and the dislocation accumulation becomes slower than that in STAGE 1. Therefore, the back-stress increment in the materials starts to decrease until the dislocations stored at the interface reach n* (STAGE 3). This relationship indicates that the accumulation of dislocations from the partitioning of TWIP-IF layered steel causes back-stress in the layered materials, and that the back-stress is saturated after sufficient dislocations occupy the TWIP-IF interface. Because of the back-stress evolution in the TWIP-IF layered steel, its strength is enhanced early in the plastic deformation process and the enhanced strength is maintained during tensile deformation. As a result, the strength of TWIP-IF layered steel is larger than the strength estimated using the rule-of-mixtures as represented in Fig. 5.

In this study, the synergetic strengthening of the TWIP-IF layered steel sheet was investigated by analyzing load partitioning behavior and dislocation density evolution using an *in situ* neutron diffraction tensile test. As a result, it was determined that three-step partitioning occurs in the TWIP-IF layered steel sheet during tensile deformation. The load partitioning at the TWIP-IF steel interface induces plastic strain incompatibility and extra-dislocation accumulation occurs in the layered steel. The *in situ* neutron diffraction tensile test reveals that the dislocation density of both TWIP-steel core and IF-steel sheath is larger than that of monolithic TWIP and IF steel. Moreover, regarding the large strain, the difference in the dislocation density between the layered and monolithic steel does not change due to the saturation of dislocations accumulated at the TWIP-IF steel interface. The extra-dislocation accumulation is correlated with the back-stress evolution, which shows that the back-stress evolves early in the plastic strain process and has little effect over the large strain. This back-stress provides extra-strength to the TWIP-IF layered steel and the strength of TWIP-IF layered steel is larger than the strength estimated by the rule-of-mixtures.

## Methods

### Sample preparation

The TWIP-IF steel layered sheet was manufactured by POSCO. To adjust the 1(IF): 2(TWIP): 1(IF) thickness stacking ratio of the layered steel sheet, the 20 mm-thick TWIP (core) steel and 10 mm-thick IF (sheath) steel plates were bonded by welding along the edge of the plates. The stacked steel sheet was homogenization treated at 1200 °C for 1 h and hot rolled from 40 to 2.5 mm thickness with a 900–1100 °C temperature regime. After the hot rolling process, the sheet was cold-rolled from 2.5 to 1 mm thickness and then annealed at 820 °C for 30 s. To compare with the layered steel sheet, the same manufacturing condition was applied to the monolithic steels.

### Mechanical testing

The mechanical properties of the layered steel sheet were evaluated using the tensile tests. The plates were machined to get 5 mm-gage length plate-type sub-sized tensile specimens. The tensile tests were conducted using a universal testing machine (Instron 1361, Instron Corp., Canton, MA, USA) at room temperature with 1 × 10^−3^ s^−1^ quasi-static strain rate. For accurate strain measurement, digital image correlation (DIC: ARAMIS v 6.1, GOM Optical Measuring Techniques, Germany) was employed with a white-black speckled pattern on the surface of the tensile specimen^35^.

### EBSD analysis

To evaluate the initial microstructure of the TWIP-IF steel layered sheets, EBSD (Hikari, EDAX, USA) analysis was conducted. The EBSD samples were prepared using mechanical (SiC paper) and colloidal (0.04 μm silica) polishing. The step size was 120 nm for each sample and a minimum confidence index (CI) value of 0.09 was set to obtain reliable results. The EBSD analysis results were post-processed using TSL OIM Analysis 7 software.

### *In situ* neutron diffraction tensile test

*In situ* neutron diffraction analysis has been conducted to measure *ε*_hkl_ and the dislocation density of the TWIP-IF steel-layered sheet during tensile deformation. The *in situ* neutron diffraction tensile test was performed at the engineering materials diffractometer (BL-19, TAKUMI) in the Materials and Life Science Experimental Facility (MLF) of the Japan Proton Accelerator Research Complex (J-PARC). The detailed information for this facility is provided in ref.^36^. Radial collimators for 3 mm gauge width was imported to cover the all of sample gauge thickness. The normal direction (ND) patterns in the axial and transversal directions were measured simultaneously using two detector banks as illustrated in Fig. S2 (See the Supplementary Material). An *in situ* tensile tests weere conducted at room temperature with a strain rate of 1 × 10^−3^ s^−1^ ^37^. The stress-strain curves from the *in situ* tensile test are represented in Fig. S3 (See the Supplementary Material). To obtain reliable data, diffraction peaks were measured for each 50 MPa spacing (during elastic deformation) and 5% elongation spacing (during plastic deformation) within 20 minutes. The diffraction peaks are represented in Fig. S4 (See the Supplementary Material). The peak position and FWHM of the diffraction peaks were fitted using Rietveld refinement with a Z-Rietveld software^38^. To exclude the instrumental effect from the diffraction peaks, the diffraction pattern of LaB_6_ standard reference material was measured and implemented during the peak profile analysis.

## Supplementary information

Supplementary Material
