# Supplementary material for: Synergetic strengthening of layered steel sheet investigated using an in situ neutron diffraction tensile test
Source: Sci Rep. 2019 May 2;9:6829. doi: 10.1038/s41598-019-43369-2 (PMC6497632; doi:10.1038/s41598-019-43369-2)
Supplement: Supplementary file 1 — Supplementary Material [file 41598_2019_43369_MOESM1_ESM.docx]

**Supplementary Material**

**Synergetic strengthening of layered steel sheet investigated using an in situ neutron diffraction tensile test**

Jung Gi Kim^1^, Jae Wung Bae^1^, Jeong Min Park^1^, Wanchuck Woo^2^, Stefanus Harjo^3^, Kwang-Geun Chin^4^, Sunghak Lee^1^, Hyoung Seop Kim^1,4,5,*^

*^1^Department of Materials Science and Engineering, Pohang University of Science and Technology (POSTECH), Pohang, 37673, Korea*

*^2^Korea Atomic Energy Research Institute (KAERI), Daejeon, 34057, Republic of Korea*

*^3^J-PARC Center, Japan Atomic Energy Agency, Ibaraki, 319-1195, Japan*

*^4^Graduate Institute of Ferrous Technology, Pohang University of Science and Technology (POSTECH), Pohang, 37673, Korea*

*^5^Center for High Entropy Alloys, Pohang University of Science and Technology (POSTECH), Pohang, 37673, Korea*

**Contents**

Supplementary Figure 1

Supplementary Figure 2

Supplementary Figure 3

Supplementary Figure 4

Supplementary Notes

Supplementary References

**Supplementary Figure 1**


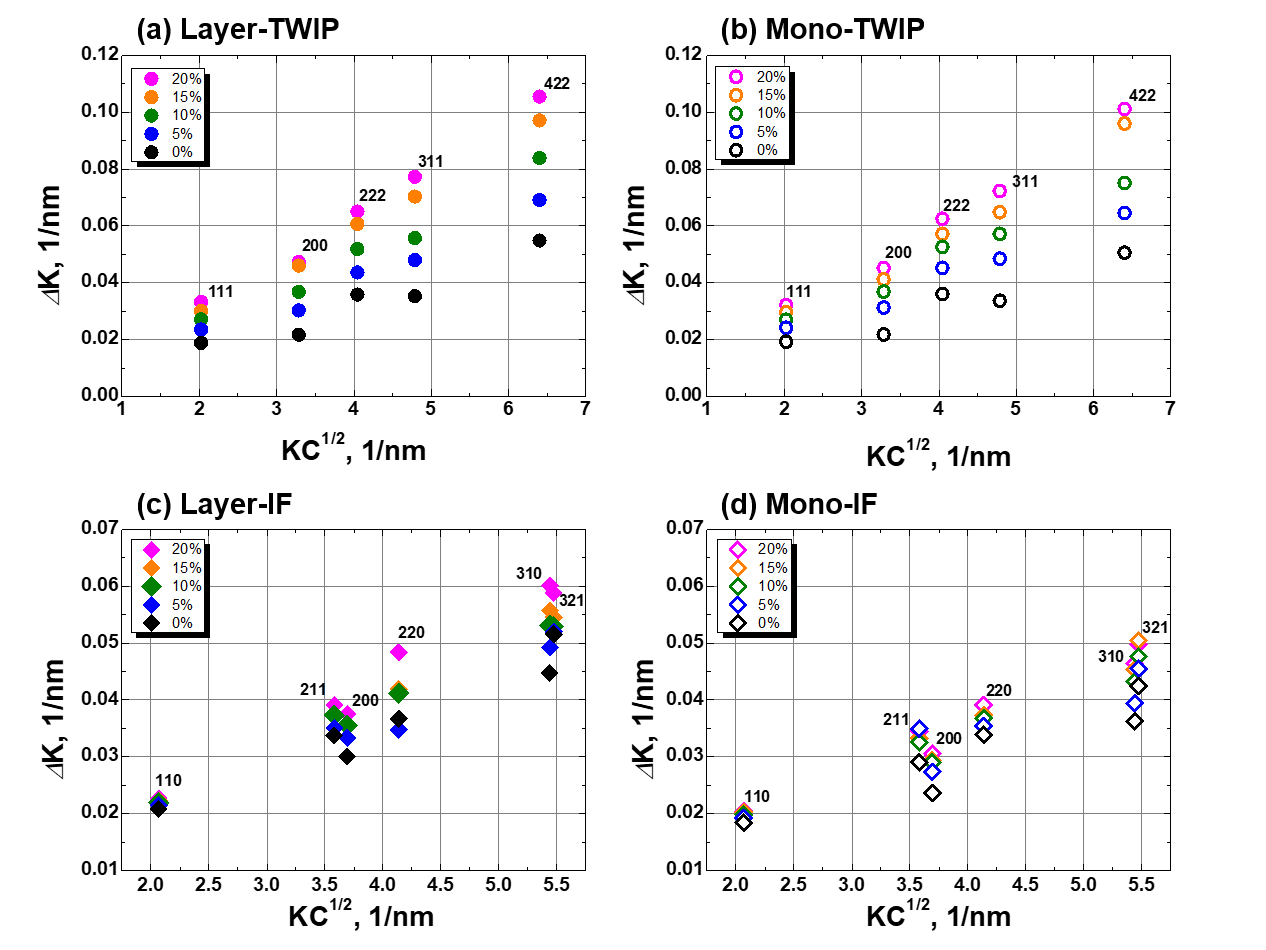


Figure S1. Modified Williamson-Hall plots of the (a) TWIP-steel core, (b) monolithic TWIP steel, (c) IF-steel sheath, and (d) monolithic IF steels.

**Supplementary Figure 2**

**
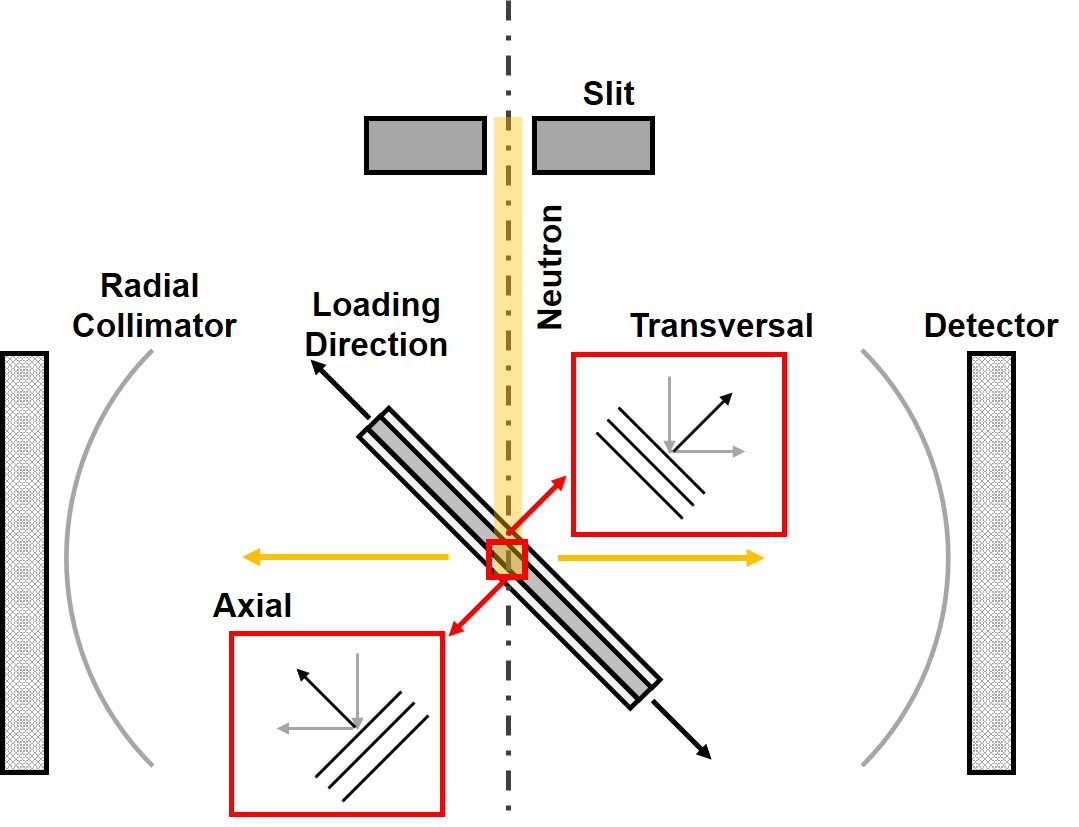
**

Figure S2. Schematic diagram of the in situ neutron diffraction tensile test at TAKUMI (BL-19), J-PARC.

**Supplementary Figure 3**


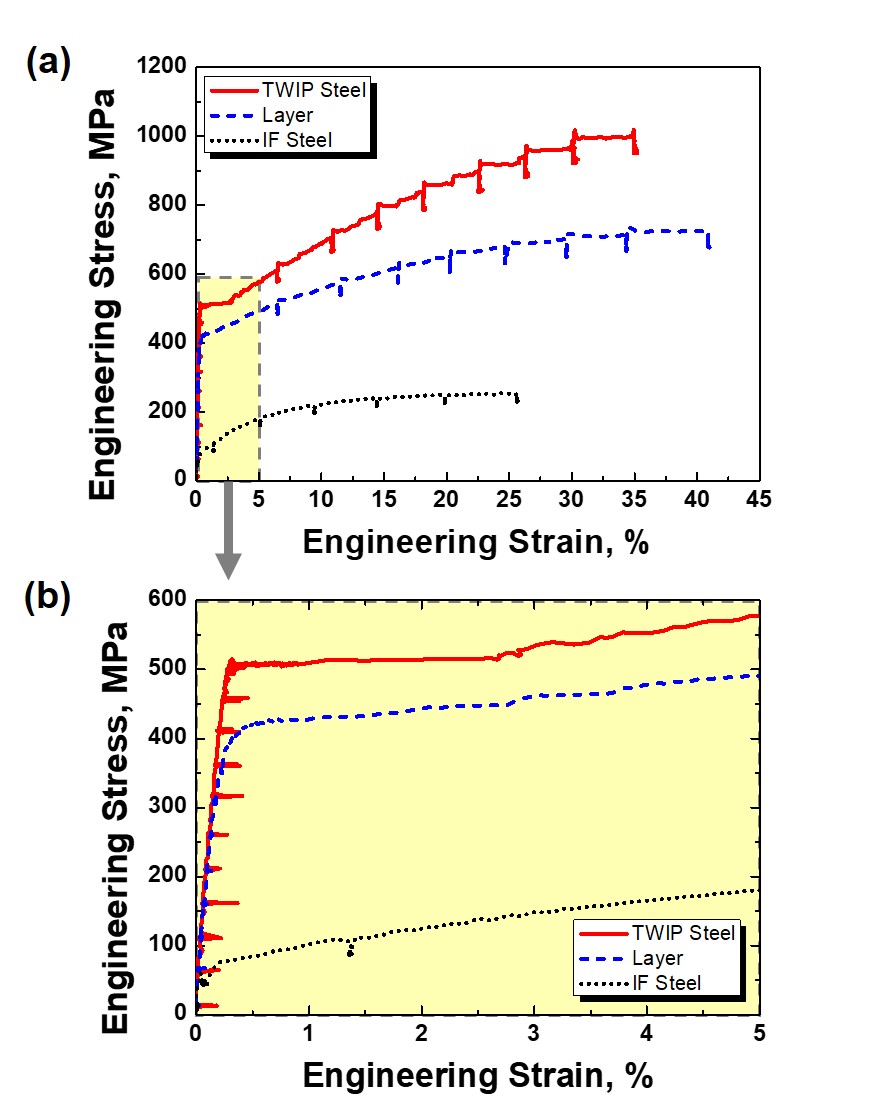


Figure S3. (a) Stress-strain curves of the TWIP, IF, and TWIP-IF layered steels from the in situ neutron diffraction tensile test, and (b) Magnified stress-strain curves in the low-strain region (elongation < 5%) of the TWIP, IF, and TWIP-IF layered steels.

**Supplementary Figure 4**

**
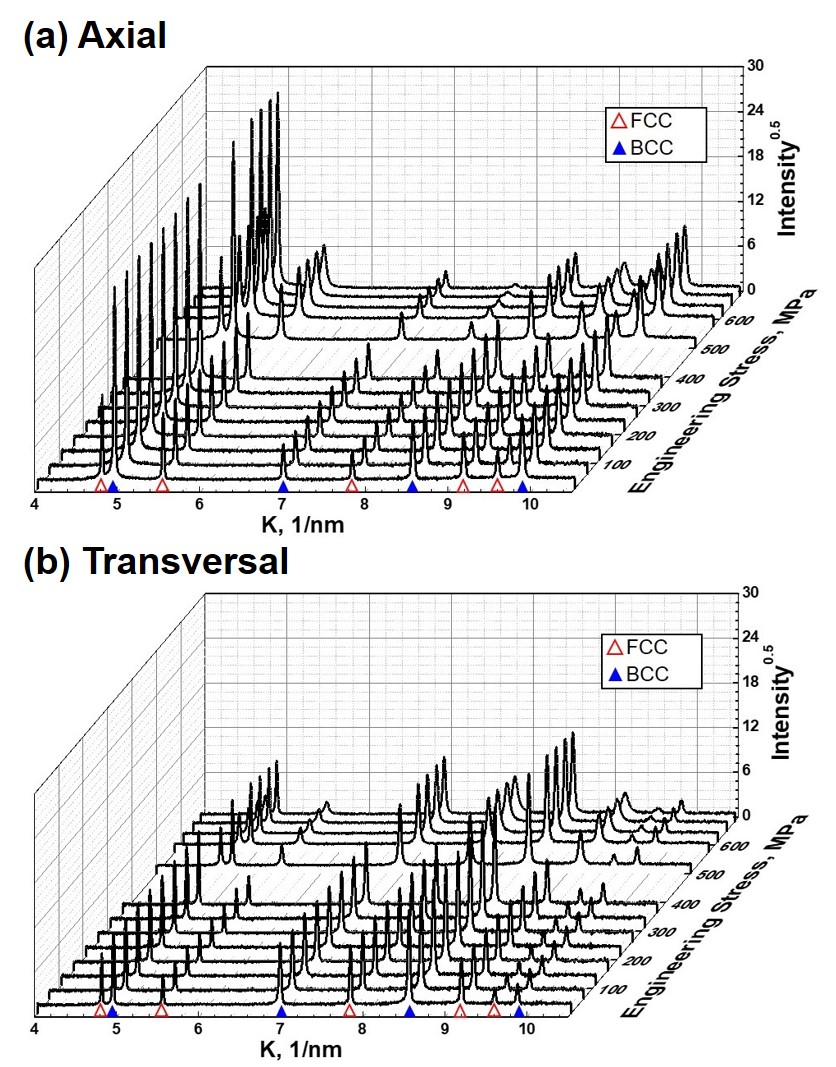
**

Figure S4. (a) Axial and (b) transversal directions neutron diffraction patterns of the TWIP-IF layered steel: Peak shift and broadening occur as the applied stress increases during the in situ neutron diffraction tensile test.

**Supplementary Notes**

**Additional explanations for Figure S3**

Figure S3 represents the stress-strain curves of the TWIP, IF, and TWIP-IF layered steels from the in situ neutron diffraction tensile tests. To measure the neutron diffraction patterns at the designated elongation, incremental-step loading was applied by modifying the crosshead displacement-control script. Because drops in the applied stress occurred while the crosshead was arrested, load drops can be observed in the neutron diffraction pattern measurement points. Although this load drop induces some experimental error during measurement, the amount of the load drop is smaller than the overall strength of the materials. Moreover, the experimental error from displacement control is smaller than that in the other control method (i.e., load control) due to the existence of large creep strain at a constant load^1^. Figure S3(b) shows the magnified stress-strain curves of the TWIP, IF, and TWIP-IF layered steels at low strain region (elongation < 5%). As mentioned in the experimental procedure of this study, the neutron diffraction patterns at the elastic deformation region were measured by controlling the applied loads. The strain deviation can be observed in the slope of the stress-strain curves due to the creep strain from the constant applied load. However, the amount of strain deviation is < 0.2% at each point and this amount of deviation is negligible during the tensile test.

**Additional explanations for Figure S4**

Figure S4 represents the axial and transversal neutron diffraction patterns of the TWIP-IF layered steel as the applied stress increases. Because the TWIP-IF layered steel was manufactured by roll bonding with face-centered cubic (FCC) TWIP steel and body-centered cubic (BCC) IF steel, both FCC and BCC diffraction peaks are observed simultaneously in the diffraction patterns. Because the applied tensile stress changes both lattice spacing and FWHM of materials^2^, the diffraction peak shift and broadening increase as the applied stress increases. From the diffraction peak shifting during tensile deformation, the *ε*_hkl_ of TWIP-IF layered steel can be calculated as shown in Fig. 2(a).

**Supplementary References**

1. Ojima, M. *et al.* Work hardening mechanism in high nitrogen austenitic steel studied by in situ neutron diffraction and in situ electron backscattering diffraction. *Mater. Sci. Eng. A* **527**, 16-24 (2014).

2. Harjo, S. *et al.* In situ neutron diffraction study of α–γ Fe–Cr–Ni alloys under tensile deformation. *Acta Mater.* **49**, 2471-2479 (2001).
